# Disease experiences and perspectives of adolescent patients with inflammatory bowel disease: a meta-synthesis of qualitative research

**DOI:** 10.3389/fpubh.2025.1696741

**Published:** 2026-01-13

**Authors:** Yue Wang, Fan Guo, Yanrong Xu, Xiaofang Feng, Yueqin Li, Liping Cui

**Affiliations:** 1 Academy of Medicine, Shanxi Medical University, Taiyuan, Shanxi, China; 2 Department of Nursing, The Third Hospital of Shanxi Medical University (Shanxi Bethune Hospital), Taiyuan, Shanxi, China

**Keywords:** adolescent, inflammatory bowel disease, experience, meta-synthesis, qualitative study, systematic review

## Abstract

**Purpose:**

The prevalence of inflammatory bowel disease (IBD) in adolescents worldwide is increasing year by year. In order to better manage the disease, it is necessary to listen to the real voice of patients. Therefore, this study aims to explore the real experience of adolescents with IBD and propose targeted intervention strategies based on the bio-psycho-social model.

**Design:**

Qualitative meta-synthesis.

**Methods:**

A systematic search was conducted across ten electronic databases—PubMed, Embase (Ovid), Web of Science, CINAHL, Cochrane Library, Scopus, PsycINFO, CNKI, Wanfang, and VIP—from their inception until October 2024. A total of 18 qualitative studies were included following a rigorous application of predefined inclusion criteria. Two independent reviewers performed study selection and data extraction, with discrepancies resolved through consensus with a third reviewer. Thematic synthesis was employed to integrate qualitative findings across the included studies in a systematic and iterative manner.

**Findings:**

The synthesized findings revealed three overarching themes and seven subthemes: (1) Challenges of self-remodeling and growth in adolescent patients with IBD under the influence of the condition; (2) The roles and function of family and healthcare systems in adolescent disease management; and (3) The complex interplay between social environmental factors and illness adaptation in adolescents with IBD.

**Conclusion:**

Adolescents with IBD encounter multifaceted experiences across individual, familial, healthcare, and social domains. Developing and implementing culturally sensitive, integrated management strategies aligned with the biopsychosocial model is essential to support these adolescents during their transition to adulthood and promote holistic development.

**Systematic review registration:**

https://www.crd.york.ac.uk/PROSPERO/view/CRD42024599200

## Introduction

Inflammatory Bowel Disease (IBD), a group of chronic gastrointestinal inflammatory disorders primarily encompassing Ulcerative Colitis (UC), Crohn's Disease (CD), and IBD-Unclassified (IBDU), presents a significant global health challenge (1). The disease course is often protracted, marked by debilitating physical symptoms (e.g., pain, fatigue, growth retardation), and often requires lifelong immunomodulatory treatment (2). While historically prevalent in Western nations, IBD incidence is now rapidly increasing in newly industrialized countries, with a notable rise among adolescents (aged 10–19 years) (3–5). This demographic is uniquely vulnerable, as the onset of IBD during this critical developmental stage can lead to delayed puberty, reduced bone density, and increased long-term cancer risks (6, 7).

Beyond the physical sequelae, the psychosocial burden on adolescents is profound. Adolescence is a pivotal period for identity formation, peer relationship development, and establishing autonomy. Chronic illness disrupts these normative processes, frequently contributing to body image disturbances, academic interruptions, strained peer relationships, heightened anxiety, and reduced treatment adherence (8–10). Consequently, adolescents with IBD report a significantly lower health-related quality of life compared to their healthy peers, highlighting an urgent need for targeted healthcare strategies tailored to their specific developmental needs (11, 12).

To develop such strategies, a deep understanding of the patient's lived experience is essential. Qualitative research offers invaluable insights into the multidimensional challenges adolescents face, including stigma, transitional anxiety, and coping mechanisms (13). However, individual qualitative studies are often limited by small sample sizes and specific cultural contexts, restricting the generalizability of their findings. Furthermore, a prior meta-synthesis addressing IBD experiences Bu et al. (14) focused predominantly on adult populations. This leaves the distinct cognitive, emotional, and psychosocial needs of adolescents—such as identity formation and autonomy in treatment decisions—inadequately synthesized.

Therefore, a systematic integration of existing qualitative evidence is necessary to provide a comprehensive understanding of this specific population. This study employs a qualitative meta-synthesis to systematically integrate findings on the lived experiences of adolescents with IBD. The synthesis aims to identify the specific biopsychosocial challenges these adolescents encounter and to inform the development of developmentally sensitive interventions.

## Methods

### Aims

This review aims to identify, explore, and synthesize qualitative evidence regarding the lived experiences and perceptions of adolescents with IBD throughout their illness trajectory and to propose corresponding management strategies.

### Study design

**Figure 1:**
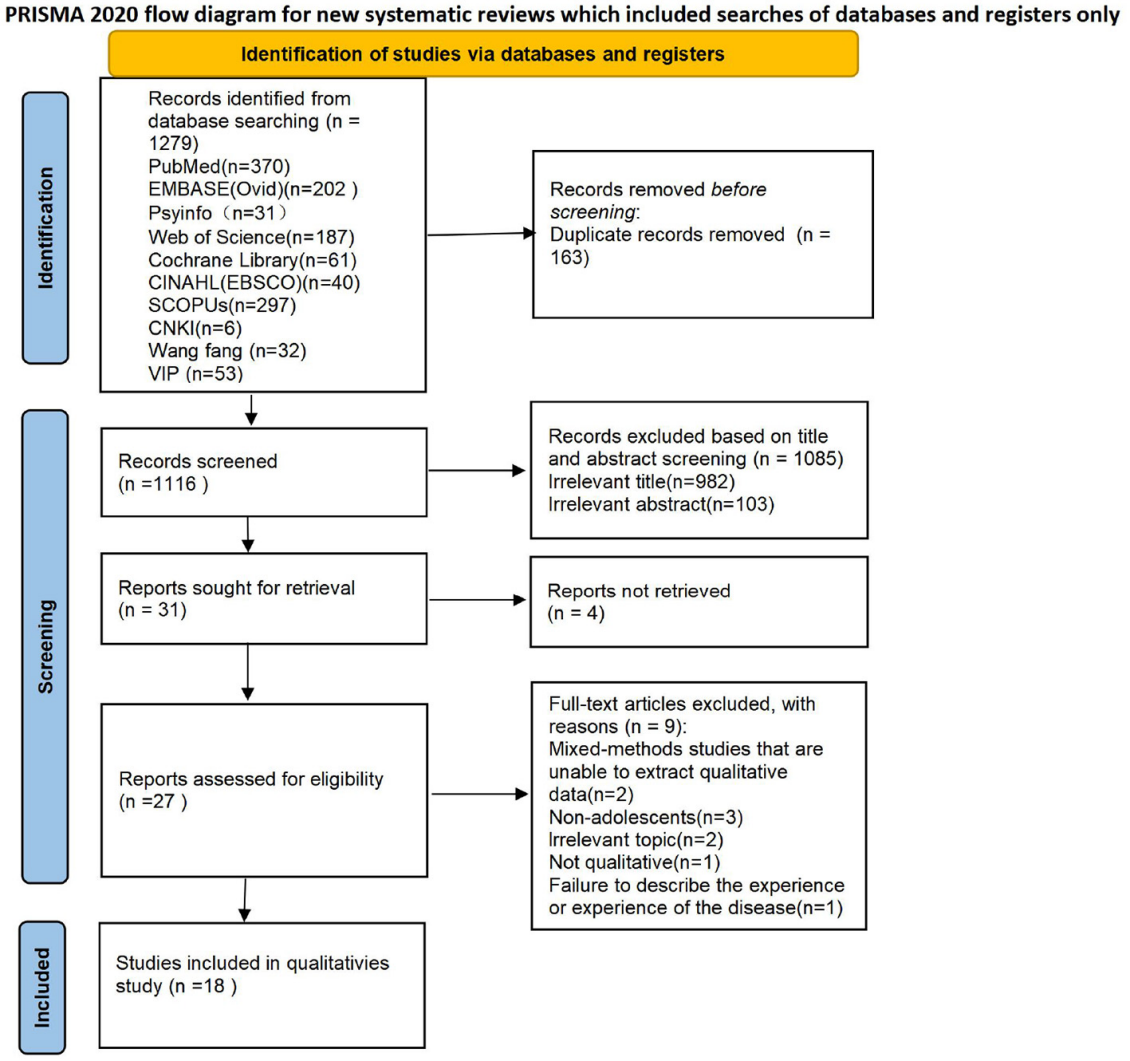
Preferred Reporting Items for Systematic Reviews and Meta-Analyses (PRISMA).

The systematic review process was conducted and reported in accordance with the Preferred Reporting Items for Systematic Reviews and Meta-Analyses (PRISMA) guidelines (15), as illustrated in Figure 1. The reporting also adheres to the 21-item Enhancing Transparency in the Reporting of Qualitative Research (ENTREQ) statement (16). Qualitative data were synthesized using thematic synthesis Thomas and Harden (13), a method that involves extracting concepts from individual studies to develop analytical themes and interpretive frameworks, while preserving the integrity of original data and researcher insights. This approach is widely used in synthesizing qualitative evidence related to healthcare experiences and perspectives (17). The study was prospectively registered in the International Prospective Register of Systematic Reviews (PROSPERO) in October 2024 (Data sheet 1, CRD42024599200).

### Search strategy

All included studies were required to meet the PICoS (Population, Interest, Context, and Setting) inclusion criteria, as detailed in Table 1. The literature search was performed following the Joanna Briggs Institute (JBI) methodological guidelines (18), which consist of three sequential steps. Step 1 involved an initial search in PubMed and CINAHL to identify relevant keywords, titles, and abstracts. Step 2 expanded the search to multiple databases, including OVID Medline, CINAHL, PubMed, Cochrane Library, Scopus, and Web of Science, across 10 English and Chinese electronic databases using both controlled vocabulary and free-text terms. Step 3 involved manual backward and forward citation searching (snowballing) of the reference lists of included studies to identify additional relevant literature. The search period covered all articles published from the inception of each database up to October 2024. The detailed PubMed search strategy and database-specific search records are documented in Supplementary File 3.

**Table 1:** Inclusion and exclusion criteria.

**Project**	**Inclusion criteria**	**Exclusion criteria**
Participants	Adolescents diagnosed with IBD (10–19 years old)	Non-adolescents, family carers, healthcare providers
Interest of phenomena Context	Feelings and experiences during illness	No experience of illness
Context	All contexts (home, hospital, community, school, etc.)	/
Types of studies	•Qualitative research (phenomenology, case studies, ethnography, narrative research, grounded theory, case studies, action research, discourse analysis, focus interviews, participant observation, field notes, content analysis, thematic analysis, etc.). •Independently extractable qualitative research components of mixed methods studies. •Article quality rated as moderate or high. •Original studies published in English and Chinese.	•Articles of non-qualitative research (e.g., quantitative studies, case studies, pilot programmes and articles of mixed studies where the qualitative part of the study cannot be extracted). •Studies for which full text is not available (e.g., conference abstracts). •Meta-synthesis or systematic evaluation. •Literature published in languages other than English and Chinese.

Two researchers independently screened titles and abstracts for eligibility. Full texts of potentially relevant studies were then independently assessed by both researchers for inclusion. Discrepancies were resolved through discussion with a third researcher, who has expertise in qualitative research methodology.

### Quality assessment

Quality assessment was conducted in three stages, as outlined by Booth (19). The first stage involved preliminary screening based on eligibility criteria. The first author applied the inclusion and exclusion criteria to identify studies meeting the minimum standards for inclusion in the review. The second stage was a technical assessment using the Joanna Briggs Institute's Critical Appraisal Checklist for Qualitative Research (JBI-CASP) (20). Two researchers independently evaluated each study using the checklist. Any discrepancies in the evaluation were resolved through discussion with a third researcher. A standardized quality evaluation form was developed to facilitate comparative analysis across studies.

The form included 10 criteria: research objectives, methodology, study design, participant recruitment, data collection methods, researcher-participant relationship, ethical considerations, data analysis rigor, clarity of results, and overall research value (results are presented in the Results section). Each criterion was assessed using a three-point scale: “Yes,” “No,” or “Unclear.” The third stage involved a theoretical assessment, evaluating the consistency between the theoretical framework and methodological approach used in each study to ensure alignment with the aims of the synthesis and the quality of the underlying theoretical foundation.

### Data extraction and data analysis

Two researchers independently performed data extraction using the Joanna Briggs Institute (JBI) Generic Information Extraction Tool. The extracted information encompassed key elements such as author, year, country, research methodology, study population, phenomenon of interest, and principal findings (detailed extraction information is presented in Table 2. Any discrepancies were resolved through discussion with a third researcher. The inclusion of studies aligned with the research objectives contributed to a deeper and more nuanced understanding of the lived experiences of adolescents with IBD.

**Table 2:** Detailed information of 18 included studies.

**No**.	**References**	**Country**	**Study design**	**Data collection method**	**Participants**	**Phenomenon of interest/Aim**	**Findings**	**Main findings**
1	Allemang et al. (21)	Canadian	Qualitative descriptive study	Virtual semistructured interviews	21 adolescent patients with IBD aged 16 to 18 years.	To explore the mental health experiences of adolescents and young adults (AYA) with inflammatory bowel disease (IBD).	AYA with IBD endorsed the criticality of incorporating mental health discussions into routine care during the transition to adult care	Three themes were generated from the data: (1) a continuum of integration between IBD and personal identity in adolescence and young adulthood; (2) manifestations of the mind-gut connection among AYA with IBD; (3) hopes and priorities for addressing mental health in IBD care
2	Suya et al. (23)	China	Phenomenological research	Semi-structured in-depth interview	15 adolescent patients with IBD aged 12 to 21 years.	To understand the dilemmas experience of adolescents with inflammatory bowel disease (IBD) as they transit toward adulthood	Adolescents with inflammatory bowel disease face multiple dilemmas in preparing for the transition to adulthood.	Four themes were extracted. Self-coping dilemma, including difficulty in adapting to changes in the medical environment, difficulty in adapting to changes in medical roles, difficulty in coping with negative emotional distress, difficulty in meeting the special needs of daily life. Family support dilemma, including heavy family financial burden, excessive participation of family caregivers, and insufficient family resilience. Medical assistance dilemma, including limited professional assistance and limited information sharing. Social support dilemma, including the limited applicability and accessibility of social support, insufficient social cognition and identity.
3	LiaoXueqin (24)	China	Qualitative descriptive study	Semi-structure interview	20 adolescents between the ages of 9 and 17.	To study the true feeling and coping experience of adolescent patients with inflammatory bowel disease (IBD).	Adolescents with inflammatory bowel disease (IBD) have a wide range of symptoms to cope with the disease.	Three themes of disease recognition, adaption to the change caused by diseases and self-psychological adjustment are extracted
4	Zhou and Huang (22)	China	Phenomenological research	Semi-structured face-to-face interviews	12 adolescents between 13 and 18 years of age.	To investigate the coping mechanisms and stress perceptions of adolescent patients with Crohn's disease.	Adolescents with Crohn's disease can better combat the condition by implementing appropriate coping strategies. Their mental health should be given attention, and a multidisciplinary team should be assembled to pro- vide them with supportive care	The 2 main themes in this study were inappropriate coping mechanisms and physical and psychological stress.
5	Chen et al. (25)	China	Qualitative descriptive study	Face-to-face in-depth interviews	14 adolescents between 14 and 17 years of age.	To explore the illness experience of adolescent patients with Crohn disease and describe the impact of the disease on the everyday lives of these individuals within the Chinese social and cultural context to provide references for targeted interventions for the healthcare team.	Healthcare providers should offer more psychological support to adolescent Crohn disease patients and advise parents to shift more attention to the mental health of their children.	Four themes were formed: (1) I am different from others, (2) I am a burden to my parents, (3) I want to be the master of my own body, and (4) I grow up suffering from illness
6	Barned et al. (27)	Canadian	Qualitative descriptive study	Semi-structured interviews	25 pediatric patients with IBD aged between 10 and 17 years old.	To better understand Children and adolescents with Inflammatory Bowel Disease (IBD) face significant and unique challenges.	young people facing great uncertainty prior to diagnosis, pronounced changes to selfhood as they make lifestyle adjustments, and facing difficulties with the implications of reduced sociability because of their disease	Three themes: challenges related to diagnosis, making sense of change, and navigating sociability.
7	Wu et al. (26)	China	Descriptive phenomenology method	Semi-structured interviews	8 adolescents aged 15 to 17 years and 24 adolescents aged 18 to 25 years were included	To better investigate how AYAs experience PTG after being diagnosed	To give tailored care to patients, medical professionals must monitor the state of their PTG development in a planned and focused manner	The interviews revealed five themes: spiritual change, internalized supportiveness, cognitive re-shaping, externalized behaviors, and future-oriented thinking
8	Wang Danyan (29)	China	Qualitative descriptive study	Semi-structured interviews	14 cases of adolescents aged 13 to 18 years.	To explore the experiences and perceptions of adolescents with inflammatory bowe disease after participating in a disease related summer camp.	The summer camp has a good effect on both disease knowledge and psychological support for adolescents with inflammatory bowel disease, thus deserving popularization.	Two themes were derived. “education is the best medicine” and “the role of powerful peers” Seven sub-themes were obtained, to improve the level of disease related knowledge, to accept the facts of illness, to increase confidence in treatment, to enhance the importance of self-management, to increase the sense of belonging. The summer camp has a good effect on both disto increase peer support, and to extend love.
9	Newton et al. (30)	America	Qualitative descriptive study	Semi-structure interview	14 adolescents (12–17 years)	To explore UC experiences in general and identify any similarities and differences in the symptoms and HRQoL impacts reported by adults and/or adolescents with UC	Open-ended interviews highlighted the HRQoL and symptomatic experiences of UC from the patient's perspective, which were similar between adult and adolescent UC patients	Only adults discussed feeling dehydrated, while only adolescents discussed the impact of UC on school life.
10	Vejzovic et al., (31)	Sweden	Phenomenological research	Individual interviews	7 cases of adolescents aged 10 to 18 years.	To illuminate the meaning of children's lived experience of ulcerative colitis	Children with inflammatory bowel disease confront various problems, such as ambitions and goals that are hard to achieve, due to reduced abilities as a result of the illness or an insufficiently adapted environment	The meaning of the children's lived experience of ulcerative colitis was summed up as a main theme. A daily struggle to adapt and be perceived as normal consisted of 4 subthemes: being healthy despite the symptoms, being healthy despite being afraid, being healthy despite a sense of being different, and being healthy despite needing support.
11	Olsen et al. (15)	Denmark	Phenomenological research	Semistructured interview	8 cases of adolescents aged 10 to 19 years.	To identify and describe adolescents' lived experiences while hospitalized after surgery for ulcerative colitis.	The findings demonstrate the importance of individualized nursing care on the basis of the adolescent's age, maturity, and individual needs.	Three themes were identified: Body: Out of order; Seen and understood; and Where are all the others?
12	Barned et al. (32)	Canadian	Qualitative descriptive study	Individual interviews	25 cases of adolescents aged 10 to 17 years.	This study sought the perspectives of Canadian children and adolescents living with inflammatory bowel disease (IBD) to determine how they go about deciding if and when to tell others about their illness.	We argue that knowledge of how children with IBD make disclosure decisions is an important part of understanding the social experience of having IBD, and in creating environments that allow them to adapt to life with IBD.	Three themes: 1. To disclose or conceal: making the decision. 2. When to tell: factors influencing disclosure decisions. 3. Challenges of IBD disclosure: the reactions of others.
13	Salazar and Heyman (33)	California	Ethnographic study	Traditional anthropological methods, such as formal and informal interviews, participant observation, and fieldnote	25 adolescents aged 8 to 18.	The aim of the present study was to investigate knowledge of pediatric patients with inflammatory bowel disease (IBD) and perceptions of Camp Gut Busters, an IBD summer camp.	Pediatric patients with IBD who attended a disease-specific summer camp benefited from the experience.	Themes are divided into two categories: (1) Themes for attending camp: “Kids Like Me” “Not the Only One” and “Perspective on IBD”; (2) Disease centered
14	Hommel et al. (34)	America	Qualitative descriptive study	Semi-structured interviews	16 adolescents and their parents	To examine adolescent patient and parent perceptions of factors that impact adherence to IBD treatment regimens using a qualitative descriptive approach.	Patients and parents experience a number of challenges related to adherence within behavioral, educational, organizational and health belief domains. Behavioral interventions should focus on these issues, reduction of perceived barriers, and effective transition of responsibility for treatment adherence.	Three themes: Factors that impede treatment adherence; Factors that facilitate treatment adherence; Additional factors related to adherence
15	Lynch and Spence (35)	New Zeeland	Phenomenological research	Semi-structured interviews	4 young New Zealanders aged between 16 and 21 years old.	To focus on discovering the youth' thoughts, feelings, and perceptions of living with Crohn disease.	The findings reveal stress as integral to living with Crohn disease. They illuminate the paradoxical relationship between fear and hope and provide insight into what helps and what hinders young people's ability to cope with the disease and its treatments.	Three themes: Stress as Integral to Living With Crohn Disease;The Paradoxical Relationship Between Fear and Hope; What Helps and What Hinders
16	Nicholas et al. (37)	Canadian	Ethnographic study	Depth interviews	80 IBD participants aged between 7 and 19 years old.	To understand the lived experience and elements of quality of life as depicted by children and adolescents with inflammatory bowel disease (IBD).	Clinical assessments need to consider the experiences and perceptions of children as they manage their IBD.	Four themes: 1. Concerns relating to IBD symptoms and treatments; 2. Vulnerability and lack of control; 3. Perceiving the self negatively as different than peer; 4. Benefits of social support; 5. Personal resources in coping
17	Reichenberg et al. (36)	Sweden	Grounded theory study	Semi-structured interviews	17 adolescents with IBD aged 12 to 18 years	The aim of this study was to identify, develop and relate concepts to describe the way in which adolescents experience and interpret their parents care and concern for them.	We found ambivalence to be the most distinctive theme to appear in the way in which these young people described how they felt about their parents' response to their disease. The clinical support for young individuals with IBD should include an awareness of the simultaneous existence of conflicting attitudes, reactions and emotions.	Four main categories emerged: ambivalence, ability/inability, compliance/resistance and trust/distrust.
18	David et al. (28)	America	Mixed method	Semi-structured interviews	8 adolescents with IBD aged 12 to 20 years	To describe the expressed educational needs of pediatric patients with IBD and caregivers in regards to ostomy surgery.	Results suggest pediatric patients with IBD have limited understanding of ostomies and limited insight into educational preferences.	Five themes: Ostomy Surgery, Preoperative Concerns, Postoperative Concerns, Education Preferences, and Social Concerns.

### Researcher reflexivity

All researchers engaged actively in each phase of the review process following training in meta-synthesis methodology. Several team members had prior qualitative research experience and had received formal training at the School of Nursing, Peking University, and the School of Nursing, Fudan University. Any uncertainties or challenges encountered during the review were discussed and resolved during weekly team meetings. To minimize researcher bias and enhance methodological rigor, potential assumptions and preconceptions were critically examined and discussed repeatedly throughout the synthesis process through structured reflexivity practices within the research team.

## Results

### Search results

As illustrated in Figure 1, a total of 1,279 articles were identified through searches in both Chinese- and English-language databases. Following the application of inclusion and exclusion criteria, 18 studies were ultimately selected for synthesis (15, 21–37). All included studies were imported into NVivo 15.0 for systematic coding and data management. Using the thematic synthesis approach proposed by Thomas and Harden (13), two researchers independently analyzed the qualitative data. Through inductive coding, three overarching analytical themes were identified. Any discrepancies between the two researchers were resolved through consensus discussion with a third researcher.

### Research characteristics

Table 3 summarizes the quality assessment outcomes of the included studies. Two studies explicitly stated their theoretical foundations, and all received ethical approval. One study fulfilled all ten quality criteria and was rated as “high” quality, while the remaining studies were assessed as “medium” quality. The detailed characteristics of the included studies are provided in Appendix 4 (Table 2). The studies originated from six countries: Canada (*n* = 4), China (*n* = 6), Sweden (*n* = 2), the United States (*n* = 4), Denmark (*n* = 1), and New Zealand (*n* = 1). Five research methodologies were represented: descriptive qualitative studies (*n* = 8), phenomenological studies (*n* = 6), ethnographic studies (*n* = 2), grounded theory studies (*n* = 1), and mixed-methods studies (*n* = 1).

**Table 3:** Quality appraisal of the included studies using the Critical Appraisal Screening Programme (20).

**Citations**	**Criteria**										**Grade**
	**Q1**	**Q2**	**Q3**	**Q4**	**Q5**	**Q6**	**Q7**	**Q8**	**Q9**	**Q10**	
Allemang et al. (21)	Y	Y	Y	Y	Y	Y	Y	Y	Y	Y	H
Suya et al. (23)	Y	Y	Y	Y	Y	C	Y	Y	Y	Y	M
Miao (24)	Y	Y	Y	Y	Y	C	Y	Y	Y	Y	M
Zhou and Huang (22)	Y	Y	Y	Y	Y	C	Y	Y	Y	Y	M
Chen et al. (25)	Y	Y	Y	Y	Y	Y	Y	Y	Y	Y	M
Barned et al. (27)	Y	Y	Y	Y	Y	C	Y	Y	Y	Y	M
Wu et al. (26)	Y	Y	Y	Y	Y	C	Y	Y	Y	Y	M
Wang Danyan (29)	Y	Y	Y	Y	Y	N	Y	Y	Y	Y	M
Newton et al. (30)	Y	Y	Y	Y	Y	N	Y	Y	Y	Y	M
Vejzovic et al. (31)	Y	Y	Y	Y	Y	N	Y	Y	Y	Y	M
Olsen et al. (15)	Y	Y	Y	Y	Y	N	Y	Y	Y	Y	M
Barned et al. (32)	Y	Y	Y	Y	Y	N	Y	Y	Y	Y	M
Salazar and Heyman (33)	Y	Y	Y	Y	Y	N	Y	Y	Y	Y	M
Hommel et al. (34)	Y	Y	Y	Y	Y	N	Y	Y	Y	Y	M
Lynch and Spence (35)	Y	Y	Y	Y	Y	N	Y	Y	Y	Y	M
Nicholas et al. (37)	Y	Y	Y	Y	Y	C	Y	Y	Y	Y	M
Reichenberg et al. (36)	Y	Y	Y	Y	Y	N	Y	Y	Y	Y	M
David et al. (28)	Y	Y	Y	Y	Y	N	Y	Y	Y	Y	M

The following 11 criteria were included: (1) Was there a clear statement of the aims of the research? (2) Is a qualitative methodology appropriate? (3) Was the research design appropriate to address the aims of the research? (4) Was the recruitment strategy appropriate to the aims of the research? (5) Was the data collected in a way that addressed the research issue? (6) Has the relationship between researcher and participants been adequately considered? (7) Have ethical issues been taken into consideration? (8) Was the data analysis sufficiently rigorous? (9) Is there a clear statement of findings? (10) How valuable is the research?

Y = yes; C = can't tell; N = no; H = high; M = moderate.

### Key findings

**Figure 2:**
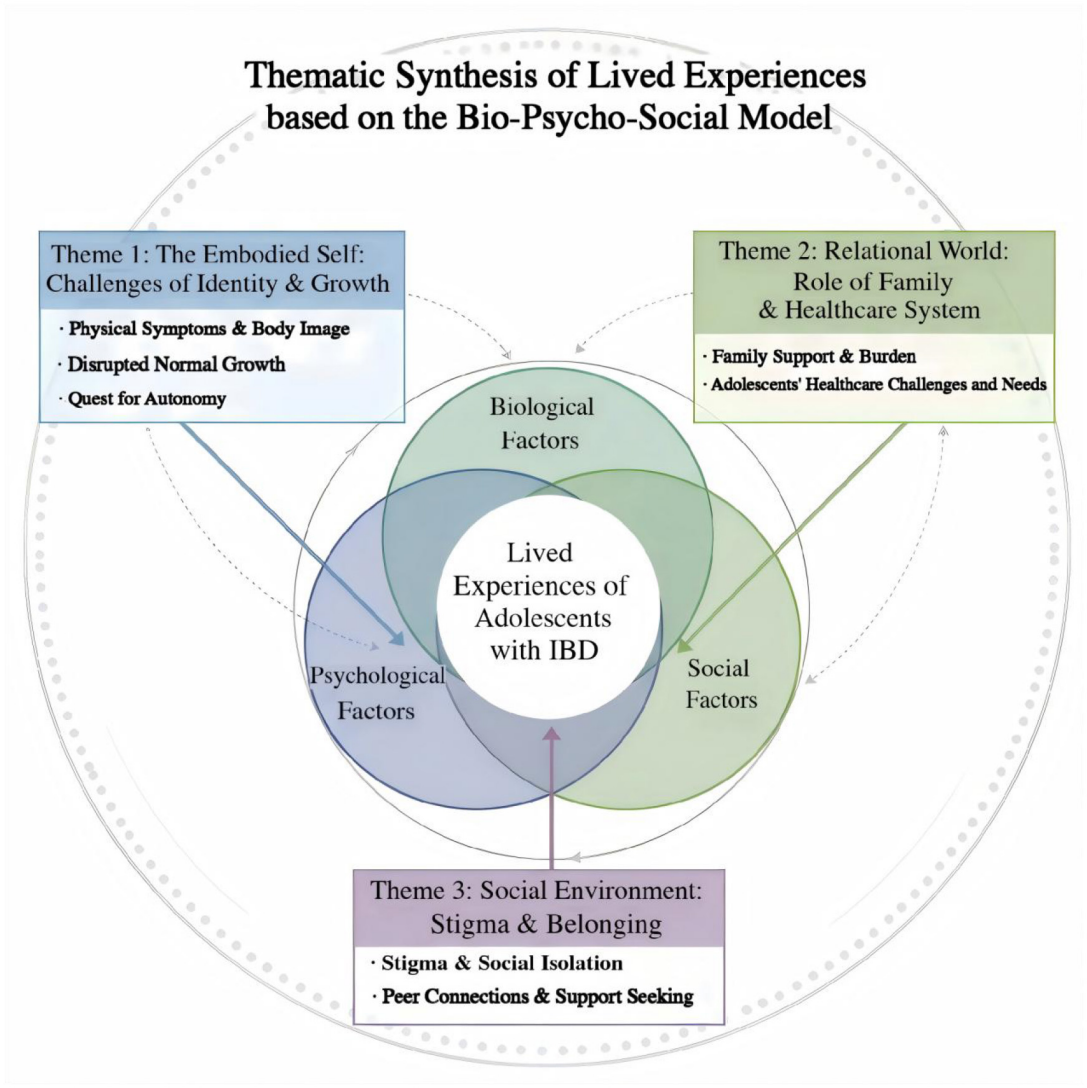
Thematic synthesis of the lived experiences of adolescents with IBD based on the bio-psycho-social model. This Venn diagram illustrates the dynamic interplay between the three overarching themes (colored circles) identified in this meta-synthesis. The intersecting areas highlight how psychological, familial/healthcare, and broader social environmental factors collectively shape the core lived experience of adolescents with IBD.

From the analysis of the 18 included studies, we identified 87 findings, grouped into 7 categories, which were synthesized into 3 comprehensive analytical themes. To provide a holistic overview of these findings within the theoretical framework, Figure 2 visually maps these themes based on the bio-psycho-social model.

Table 4 outlines the synthesized themes along with illustrative quotations.

**Table 4:** Thematic results of integration and example application.

**Analytic themes**	**Descriptive themes**	**Quotations from participants in primary study**
Challenges of self-remodeling and growth in adolescent patients with IBD under the influence of the condition.	The Process of Self-Cognition Reconstruction and Psychological Growth under Disease Awareness and Self-Perception	•“I'm Not a Normal Healthy Teenager” “So, in those kinds of years, it kind of puts you behind everybody else.” (S-034) (21) •My abdominal pain wouldn't make me feel inferior, but the differences would … When i was diagnosed with CD, i was told to avoid strenuous exercise, so my physical strength was worse than others. Then, my grades were poor, and i felt that i was a useless person. (p12) Now, i think that this disease isn't a very serious thing. (i) only need to take medicine and control my diet. it doesn't have much impact (on me). (This disease) isn't as scary as what was shown on the internet, and i just think that i could manage it on my own. (p10) (25) •“I admit that I have been in a negative mood at times. Compared to those in a worse situation than mine, or when I heard about some unfortunate events, I found that I was doing quite well”. (26) •(Paddy)Once I had my surgery my attitude changed completely...I crossed over the wall. It was very hard at first...the ileostomy came as a big shock. You have to have an open mind... (35) •“I am so different because I have a strange disease” (37)
	Complex Psychological States and Adaptive Mechanisms in Illness Management	•This led AYA to feel isolated, “invalidated,” and “down” about “life being sick” (S-030) (21) •There was several times when I said, “Let me just have the final jab and then it's over, because I can't take any more.” I did not want to be here any longer because I felt so bad and it just hurt. (Informant 7) (15) •I think it it is kind of like umm it is not really something that you really want to talk about but it is like something it is like kind of something it is a topic you'd much rather avoid when you talk to people with you'd if I could if I I prefer not to tell very many people about it. (32) •(As a result of going to camp) I didn't feel isolated. I didn't feel like I was alone. (33) •“I never know when I'm going to get fat again. It's awful”; “You never know what is going to happen” (37)
	Social Contextual Demands and Coping Strategies.	•N8: “I'm scared to talk to my friends, my old best friends are hardly in touch now, I just want to stay at home and nobody should bother me.” (23) •P-18: “When I knew there was no cure for IBD, my world collapsed. I locked myself in the room every day and didn't want to face it”. (26) •N8: I'm fat after taking hormones, and my classmates will say I am. N10: “My classmates make fun of me for repeating a grade and say I'm still in the fourth grade.” (24) •P4: “During class, I often feel compelled to go, and it's embarrassing because I sense that my classmates are giving me odd looks whenever I do.” (22) •p8: I couldn't attend some social activities and parties. Things like communication or hanging out with my friends had become less and less frequent, and i felt as if i was estranged from my classmates to a large extent. M10: “I just don't feel as lonely as I used to, I feel like a lot of people have this disease, I feel like I've found an organisation.” (25) •Peggy: I have a good 5–6 friends who were always supportive. They would come with me to appointments. (35)
The role and function of family and medical systems in adolescent disease management.	Complex emotional and behavioral dynamics within family relationships of adolescent patients.	•N1: I didn't need to prepare for the transition from paediatrics to adult unit because my parents were there and I didn't have to worry, I just listened to my parents, doctors and nurses. N5: I feel like a big pain in the arse, bothering my parents all the time, and they must hate me too, right? (23) •N19: Families are financially burdened and it takes a lot of time, money and effort. N2: Mum searches on the Internet and I look at what is said on the Internet with Mum. N17: Mum reads books and watches TV to learn about the disease, and I look at what I can eat with her. (24) •p11: I knew their intention was good, but i didn't want to get too much attention. i felt like i was treated as “a national treasure”. i didn't want them to act like that. I just wanted to tell them to treat me as usual; otherwise, it would impose more burden to me. (25) •P27: I did not know well about IBD, but my parents' reaction to the disease scared me. (26) •Informant 4: “I know what a bloody nuisance one's parents can be, but after all, they do know how their child is doing, and how they prefer things to be done, so I was extremely happy that they were with me” (15).
	Multidimensional challenges and healthcare needs experienced by adolescents in medical service encounters.	•S-020 “I feel like maybe in the future, I think [a transition navigator] is something that would benefit everyone a lot. Because there's so many questions and things that I would not have even known about or I would have been stressed about because I wasn't sure how to move to adult care.” (21) •N8: I was hospitalised once or twice before and had to have a colonoscopy.I was scared, didn't know what it was, didn't like laxatives, and had never heard of enemas, so I was afraid and resisted. (24) •(Informant 7) “But those cross and cold nurses … it was mostly the older ones … they'd say ‘There, it's like this and like that”. (Informant 4) “She was just so cold … she had no feelings whatsoever” (15) •P11: “I visited multiple hospitals and even consulted a Chinese medicine practitioner and tried Chinese remedies, but I didn't experience clear improvement, and my stomach pain persisted, ultimately leading me to seek help at a hospital.” (22)
		•N5: I didn't know what I needed to do to prepare, or when I should start preparing, and it wasn't clear whether to pause or continue treatment when I encountered problems, and I didn't have a professional to guide me through the transition. (23) •“Probably written down so I can read it and that's how I take things in so I can look at it.”. “I would like to have both [written and verbal] at least and also like face to face.” (28)
The intricate interplay between social contextual factors and illness adaptability in adolescents with IBD.	The Multidimensional Impact of IBD and Adolescents' Adaptive Coping Strategies.	•CH-012 “...It's kind of like a sharp, sharp pain...and just...It's pain...right here on my abdomen...That area.” (29) •p7: I suddenly found that i have become different. (25) •P3: “Undoubtedly, the greatest impact is on my academics, and frequent hospital visits eat away at my precious time.” (22) •S-027 “I get stressed often, and then I do feel like I have more symptoms if I'm feeling stressed”. S-013: “In terms of IBD impacting my mood, I would say it was when I was in my flare. It took so much energy out of me, I couldn't do anything, I was just lying in bed basically all day.” (21) •N9: “The new access environment (adult healthcare system) was very different from paediatrics, [adult unit healthcare staff] were more concerned about changes in my condition and didn't approve of parental intervention, which made it difficult for me to adapt for a short period of time.” (23) •P2: “I know my mood is volatile; I can only do the best to adjust; watching TV is a better effective strategy to stop thinking”. P19: “Once I get into a state of self-entanglement, I will force myself not to think or find something else to do to divert my attention” P8: “I want to make myself feel better. Because I found that mood is magical, when I am in a good mood, my bowel symptoms will improve accordingly. My doctor also told me that they were related”. P20: “I used to put my career first, giving up my health for work. It's not too late to realize that health is the most important thing” (26)
	Social Environmental Barriers and Resource Utilization Patterns.	•N4: “Sometimes I have to poop dozens of times in 1 d. I often go to places where there are not always bathrooms, and I can't get there in time when the urge to poop comes.” N13: “Biological agents need to be used regularly, but they are more expensive, with high out-of-pocket expenses, there is no way to buy commercial insurance for this disease, there is no corresponding supportive policy, and I have limited social support.” (23) •“And I didn't really how to handle between that and school.” (S-031). “When I was sick it was very frustrating, not being able to go out or being too scared to go out. I remember a couple of times I went to [amusement park] with my family and I wasn't even able to make it to the front of the park without needing to go to the bathroom. I remember my dad had to carry me and as a little bit of an older kid, I was in the fifth or sixth grade, but it's a little embarrassing I'm not gonna lie. It's definitely hard.” (S-030) (21) •AIDS everybody knows about. But when have you seen a commercial that says “help people with ulcerative colitis?” (33) •“I'm thinner, pale, my hair is thinner. I don't like how I look now. I look sickly”; “Everyone else is growing, but I'm shrinking”; (37) •They put me on prednisone and I had a reaction. It made my whole body blow up. I put on so much weight and my face went all round…. It changes you r whole look. (Amy). I was really scared of coming out with a bag.... I love swimming and surfing at the beach so that was huge worry.... I still have ongoing crap... the usual toilet dramas, but not as often. (Amy). (35)

Description: This table presents the main themes, sub-themes, and the quoted statements from the respondents that were integrated in this study.

#### Comprehensive finding 1: challenges of self-remodeling and growth in adolescent patients with IBD under the influence of the condition

This overarching theme encompasses three interrelated sub-themes: (1) The Process of Self-Cognition Reconstruction and Psychological Growth under Disease Awareness and Self-Perception; (2) Complex Psychological States and Adaptive Mechanisms in Illness Management; and (3) Social Contextual Demands and Coping Strategies.

(1) The Process of Self-Cognition Reconstruction and Psychological Growth under Disease Awareness and Self-Perception

The experience of illness initiates a process of self-cognitive restructuring, prompting adolescents to reflect on their self-image, identity, personal values, and future aspirations. This dynamic transformation reflects how adolescents reframe their understanding of self in the context of chronic illness. Cognitive restructuring serves as a foundational mechanism for coping with the disease and rebuilding a coherent sense of identity. Within this process, adolescents exhibit a dual emotional trajectory: on one hand, disease uncertainty and bodily dysregulation can trigger episodic emotional distress:

*I get scared that something is going to happen to me …. I don't want them to cut me open. I'm scared something is gonna go wrong with the operation and after the recovery…. I have a lot of fear of being in extreme pain and having an operation. (Sally)—(34)*

“*IBD makes me feel frightened... I feel like I have no control over my own body whatsoever.” “I'm often feeling ill, then its hard to eat, get up and concentrate”; “I never know when I'm going to get fat again. It's awful.—(35)*

On the other hand, many adolescents demonstrate post-traumatic growth through meaning-making (e.g., “the disease made me appreciate life more”) and upward social comparison (e.g., “I'm still fortunate compared to those with more severe conditions”).

“*I admit that I have been in a negative mood at times. Compared to those in a worse situation than mine, or when I heard about some unfortunate events, I found that I was doing quite well.”*

“*My life has changed since I got sick. Every person has only one life, and I want to live it better.”*

“*It made me realize that I had to live for myself... My life needed variety,”—(26)*

(2) Complex Psychological States and Adaptive Mechanisms in Illness Management

Notably, cognitive restructuring plays a pivotal role in emotion regulation. Adolescents who develop a coherent understanding of their illness are more likely to engage in adaptive emotional expression. This development of psychological resilience is closely linked to both disease awareness and self-perception, and it influences their functioning in familial, social, and daily life contexts. These internal psychological resources provide the motivational foundation for adolescents to re-engage with social systems and reconstruct their roles within them.

*Once I had my surgery my attitude changed completely... I crossed over the wall. It was very hard at first … the ileostomy came as a big shock. You have to have an open mind...so generally I tend to keep it quiet. (Paddy)—(34)*

“*So, I mean, I can get through days without thinking about it, but sometimes I just reflect on my life, and I'm like, wow, this is my life, you know?” (S-012)—(21)*

*I used to care about who disliked me and many things like this...Now, i feel that it isn't so important; health is the most important thing. (p7)—(25)*

*Participant 9: “It is not easy for someone who has IBD. I am willing to provide emotional counseling and disease guidance to people who have just been diagnosed.”—(26)*

(3) Social Contextual Demands and Coping Strategies

Adolescents' social experiences encompass a range of interpersonal challenges, including difficulties in peer relationships, the need for friendship and social support, behavioral adaptations in social settings, and the broader psychological and disease-related impacts of social relationships. These experiences highlight the multifaceted nature of adolescents' social needs and challenges. Social interaction influences the self-reconstruction process through two distinct pathways:

Supportive Pathways: Peer support enhances self-esteem; collaborative patient-provider relationships improve treatment adherence; and evolving family roles foster a sense of personal responsibility.

*Instead of my healthy friends, fellow patients of a similar age could better understand and empathize with me. Because we were peers, we suffered from the same disease, and we shared similar topics. (p14)—(25)*

*When I was diagnosed, I was pulled into a chat group, a group of wardmate. I felt like I'd found my place. They described their experiences and made me feel strong enough to live.—(26)*

Stressful Pathways: Social withdrawal may lead to progressive social disengagement, while perceived stigma exacerbates self-stigmatization and identity fragmentation. There is a strong positive association between an individual's social adaptability and their level of cognitive and emotional preparedness prior to encountering social transitions or stressors. As revealed in qualitative accounts, a key mechanism through which social interaction facilitates personal growth involves the reinforcement of self-efficacy and identity coherence.

*Staying at home alone for such a long time gradually made me very self-contained and closed off. Due to my suspension of school, it seemed as if there were fewer things that i could talk about with my classmates. I felt that we couldn't get along as before. (p14)—(26)*

#### Comprehensive finding 2: the roles and functions of family and healthcare systems in adolescent disease management

This comprehensive finding comprises two interrelated sub-themes: (1) Complex emotional and behavioral dynamics within family relationships of adolescent patients, and (2) Multidimensional challenges and healthcare needs experienced by adolescents in medical service encounters.

(1) Complex Emotional and Behavioral Dynamics Within Family Relationships of Adolescent Patients

The family environment and relational dynamics significantly influence adolescents' psychological wellbeing, disease management capabilities, and self-identity development. These influences are shaped by parental parenting styles, family atmosphere, and patterns of interaction among family members. Secure attachment relationships have been shown to reduce anxiety levels in adolescents. However, parenting styles characterized by high emotional involvement may impair adolescents' autonomy and decision-making abilities. Furthermore, family illness narratives—such as how the family collectively understands and talks about the disease—can positively predict adolescents' self-efficacy. Positive family relationships are also associated with higher treatment adherence. Conversely, the transmission of parental distress or burden significantly intensifies adolescents' psychological stress.

*N2: Mummy searches on the internet and I read what the internet says together with her. N17: Mummy reads books and watches videos to learn about this disease, and I look at what I can eat with her. — (24)*

Research also reveals intergenerational transmission effects, wherein parental coping strategies in response to illness can shape and partially explain the disease management approaches adopted by their adolescent children. This suggests that family systems play a dual role as both a source of support and a potential contributor to disease-related stress in adolescents.

“*I was so discouraged to see my parents always sighing and wishing that the family could sit together and talk about it instead of keeping their mouths shut, and now even my relatives and friends don't dare to enquire about my illness.”?—(23);*

*Participant 27: I don't know much about IBD, but my parents'reaction to the disease scares me.— (24)*

(2) Multidimensional Dilemmas and Healthcare Needs of Adolescent Patients in Medical Service Encounters

The experiences and perceptions of adolescent patients throughout the healthcare process reveal multiple dimensions of challenges and unmet needs. These include accessibility and convenience of medical services, the attitudes and professional competence of healthcare providers, the effectiveness of clinical guidance, and the degree of integration and coordination within the healthcare system. Collectively, these factors reflect both the expectations adolescents hold toward healthcare support and the systemic shortcomings that currently exist.

High-quality healthcare services have the potential to significantly enhance adolescents' understanding of their condition and strengthen their confidence in managing the disease. For example, empathetic care and evidence-based guidance from healthcare professionals have been shown to improve medication adherence and psychological wellbeing. In contrast, deficiencies in healthcare delivery—such as fragmented communication, lack of adolescent-friendly services, or insufficient psychosocial support—can exacerbate feelings of fear and helplessness, which may subsequently strain family dynamics.

Synthesis of the evidence suggests that when family emotional resilience and supportive healthcare environments interact synergistically, there is a measurable improvement in both treatment adherence and overall quality of life among adolescent patients.

*But those cross and cold nurses … it was mostly the older ones … they'd say ‘There, it's like this and like that”. (Informant 4) “She(nurse) was just so cold … she had no feelings whatsoever”— (31)*

*N19: I wish there were more dietary guidance classes to tell us specifically if we can eat yoghurt, milk and some other things and how to eat them.— (24)*

#### Comprehensive finding 3: the complex interplay between social environmental factors and disease adaptation in adolescent IBD patients

This comprehensive finding consists of two interrelated sub-themes: (1) The Multidimensional Impact of IBD and Adolescents' Adaptive Coping Strategies, and (2) Social Environmental Barriers and Resource Utilization Patterns.

(1) The Multidimensional Impact of IBD and Adolescents' Adaptive Coping Strategies

This sub-theme illustrates the wide-ranging negative effects of IBD across physical, psychological, academic, and daily life domains. In response to these challenges, adolescents actively engage in adaptive efforts, such as modifying their mindset, developing coping strategies, and adjusting lifestyle routines to manage disease-related disruptions. These adaptive behaviors are shaped by and interact with the broader social environment. For example, academic difficulties caused by the disease often prompt adolescents to seek external support or reevaluate their educational goals. This dynamic process highlights the reciprocal relationship between individual coping and environmental influences, which ultimately shapes the effectiveness of disease adaptation.

*P3: “Undoubtedly, the greatest impact is on my academics, and frequent hospital visits eat away at my precious time. I constantly have to request time off, leaving me unable to catch up on missed classes. It's quite disheartening, and I fear my classmates may outperform me in the upcoming monthly exam. Sometimes, I struggle to fall asleep until the early morning hours.— (24)*

*P: Colitis affects my liver. I can't drink alcohol so that presents, well, it's not really a problem, but I can't do the stuff that some of my friends are doing. I can't, I won't ever be able to drink alcohol.—(27)*

“*Meditation and breathing techniques edall of these things have taught me when I'm in a frustrating or stressful situation, even if it's not with my illness, I feel like I can use those techniques not only with my illness but just in my day-to-day life so that I can be comfortable in any situation that I'm in.” (S-019)— (21)*

(2) Social Environmental Barriers and Resource Utilization Patterns

Social environmental factors significantly influence adolescents' disease experiences through multiple pathways, including sociocultural norms (e.g., perceptions of body image), support systems (e.g., policy frameworks and community assistance programs), and infrastructural accessibility (e.g., availability of public restroom facilities). These factors affect patients' quality of life, psychological wellbeing, and capacity to manage their condition. Furthermore, adolescents express clear needs and expectations regarding accessible and inclusive social resources. For instance, societal stigma surrounding chronic illness can lead to social withdrawal, heightened psychological distress, and increased internalized shame. These experiences underscore the dual role of the social environment as both a source of adversity and a potential facilitator of adaptive outcomes.

*People don't understand. Cancer—everybody knows about. AIDS everybody knows about. But when have you seen a commercial that says “help people with ulcerative colitis?”—(32)*

“*I've been called ‘cancer boy' because of thin hair”; “Kids make fun of my problems”; “A group of people push me around.”—(35)*

*N13: Biological agents need to be used regularly, but they are more expensive, with high out-of-pocket expenses, there is no way to buy commercial insurance for this disease, there is no corresponding supportive policy, and I have limited social support. I have limited social support. N4: Sometimes I have to poop dozens of times in 1 d. I often go to places where there are not always bathrooms, and I can't get there in time when the urge to poop comes. —(23)*

### ConQual summary of the synthesized findings

The ConQual evidence grading method was used to evaluate the quality of evidence of the integration results. The quality level of the evidence body of qualitative studies integration results follows the GRADE system. No matter what type of qualitative study is integrated, the starting point of the evidence body quality level after integration is “high.” According to the reliability and credibility evaluation of the integration results, the quality level of the evidence body was judged to be downgraded, and the quality level of the evidence body was formed.

Table 5 presents the ConQual evidence grading results.

**Table 5:** ConQual summary of the findings.

**Synthesized findings**	**Type of research**	**Dependability**	**Credibility**	**ConQual score**
Self-reinvention and growth challenges of adolescents with IBD under the impact of the disease.	Qualitative	High	Downgrade 1 level	Medium
The roles and functions of the family and the healthcare system in the management of the disease in adolescents.	Qualitative	Downgrade 1 level	No change	Medium
The complex relationship between socio-environmental factors and the adaptation to the disease in adolescents with IBD.	Qualitative	Downgrade 1 level	Downgrade 1 level	Low

## Discussion

This meta-synthesis provides a comprehensive framework for understanding the lived experiences of adolescents with IBD. By integrating findings into the biopsychosocial model proposed by Engel (38), our results confirm that health outcomes in this population are not solely determined by biological inflammation but emerge from the dynamic interplay between physiological vulnerability, psychological adaptation, and social context. Our synthesis addresses the theoretical gap in previous reviews by explicitly mapping how these domains interact specifically during the critical developmental stage of adolescence.

The Interplay of Biology and Psychosocial Development Adolescence is a pivotal period for identity formation [Erikson's stage of “identity vs. role confusion” (39)]. Our findings reveal that chronic physical symptoms (pain, fatigue, delayed puberty) fundamentally disrupt this normative process, creating a “dual identity conflict” between being a “patient” and a “normal peer.” Unlike adult patients, adolescents often lack the cognitive resources to integrate these conflicting identities, increasing vulnerability to role confusion and subsequent psychosocial crises. However, consistent with post-traumatic growth theory (26), many adolescents also demonstrate resilience through meaning-making strategies (e.g., “the disease made me stronger”), suggesting that interventions should not only focus on symptom reduction but also on facilitating these adaptive cognitive reappraisals.

Sociocultural Influences on Disease Experience Our synthesis highlights significant cross-cultural divergence in how IBD is experienced (40), particularly between Eastern (e.g., China) and Western contexts. While Western adolescents often prioritize autonomy and fear social isolation from peer activities, Chinese adolescents report unique stressors deeply rooted in Confucian family values and intense academic pressure (41, 42). They often experience profound “economic guilt” due to the high cost of biologics and may adopt passive roles in decision-making to demonstrate filial piety (43, 44). Furthermore, cultural stigma related to “face” may lead to more covert coping strategies in Asian cultures compared to the more open disclosure often encouraged in Western support systems. These findings underscore that clinical guidelines cannot be “one-size-fits-all”; interventions must be culturally adapted.

Bridging the Gap: From Theory to Clinical Support Currently, a disconnect exists between adolescents' complex psychosocial needs and the fragmented care they often receive. The reliance on maladaptive coping strategies [e.g., social withdrawal to avoid stigma (45)] is frequently exacerbated by a lack of developmentally appropriate professional guidance. Effective management requires shifting from a purely biomedical focus to a holistic approach that includes routine psychosocial screening [given that adolescents with IBD are 4.6 times more likely to experience depression and anxiety (46)], structured transition programs that promote self-management autonomy, and therapeutic interventions such as Cognitive Behavioral Therapy (CBT) (47–49) and peer-led narrative interventions (50).

## Limitations

First, restricting the review to English and Chinese literature may have excluded relevant cultural perspectives from other non-English speaking regions (e.g., Latin America, Middle East). Second, the primary studies varied significantly in methodology (phenomenology vs. descriptive qualitative) and quality, with most rated as moderate quality, potentially affecting the depth of the synthesized findings. Third, our synthesis did not granularly differentiate between early (10–14 years) and late (15–19 years) adolescence, despite likely differences in their developmental needs and coping capacities.

## Future research

Future research must move beyond descriptive studies to address critical gaps in evidence-based interventions: (1) Longitudinal Transition Studies: Employ longitudinal qualitative or mixed-methods designs to track how adolescents' coping strategies and identity constructions evolve as they transition from pediatric to adult care systems. (2) Intervention Development and Testing: Shift focus from merely identifying needs to developing and rigorously testing biopsychosocial interventions. Examples include digital therapeutics for real-time stress management, peer mentorship programs to improve transition readiness, and culturally adapted family therapy protocols. (3) Health Policy and Systems Research: Investigate the impact of macro-level factors, such as different medical insurance schemes and educational policies, on the long-term psychosocial outcomes of adolescents with IBD, to inform more inclusive public health policies.

## Data Availability

The datasets presented in this study can be found in online repositories. The names of the repository/repositories and accession number(s) can be found in the article/Supplementary material.
