# Supplementary material for: Disease experiences and perspectives of adolescent patients with inflammatory bowel disease: a meta-synthesis of qualitative research
Source: Front Public Health. 2026 Jan 13;13:1696741. doi: 10.3389/fpubh.2025.1696741 (PMC12838276; doi:10.3389/fpubh.2025.1696741)
Supplement: Supplementary file 1 [file Data_Sheet_1.pdf]

NIHR | National Institute for Health and Care Research

PROSPERO
International prospective register of systematic reviews

Disease experiences and perspectives of adolescents with inflammatory bowel disease: A systematic review and meta-synthesis

Yue Wang, Lipin Cui, Xia Cheng

To enable PROSPERO to focus on COVID-19 submissions, this registration record has undergone basic automated checks for eligibility and is published exactly as submitted. PROSPERO has never provided peer review, and usual checking by the PROSPERO team does not endorse content. Therefore, automatically published records should be treated as any other PROSPERO registration. Further detail is provided here.

Citation
Yue Wang, Lipin Cui, Xia Cheng. Disease experiences and perspectives of adolescents with inflammatory bowel disease: A systematic review and meta-synthesis. PROSPERO 2024 Available from https://www.crd.york.ac.uk/PROSPERO/view/CRD42024599200

REVIEW TITLE AND BASIC DETAILS

Review title
Disease experiences and perspectives of adolescents with inflammatory bowel disease: A systematic review and meta-synthesis

Review objectives
What are the experiences and experiences of adolescents with inflammatory bowel disease during their illness? What recommendations can be derived from these included studies for further clinical practice, education and research?

Keywords

Adolescents, inflammatory bowel disease, qualitative research, Synthesis of qualitative studies

SEARCHING AND SCREENING

Searches

A search of the literature will be conducted in twelve data-bases，0VIDMedline, Psyinfo, CINAHL, Embase, Web of Science, Cochrane Library, PubMed, JBI (Joanna Briggs Institute), Scopus, China Knowledge Network, Wanfang, Sinomed.Current to October 2024, keywords and search term combination will be used for the following concepts:Adolescents; inflammatory bowel disease; qualitative research; Synthesis of qualitative studies; experiences; experience.The search will be limited to human trials.

Study design

Qualitative studies. Mixed-method studies will be considered for inclusion in the review, if the qualitative information is separately extractable and clearly identifiable.

ELIGIBILITY CRITERIA

Condition or domain being studied

Inflammatory bowel disease, a group of autoimmune disorders characterised by inflammation of the small and large intestines, in which the digestive system is attacked by the body's autoimmune system, includes Crohn's disease and ulcerative colitis, in which patients experience abdominal symptoms such as diarrhoea, abdominal pain, bloody stools and vomiting. There are 1.3 billion adolescents in the world today, representing more than one-sixth of the global population. Currently, the incidence of IBD in adolescents is on the rise globally, with a 22.8% increase in the incidence of IBD in children and adolescents globally from 1990 to 2019. Adolescents with IBD may also have comorbidities, which can have a serious impact on the quality of life of adolescents, and the feelings of the adolescent population can be easily overlooked. Quantitative and qualitative studies have been conducted on such patients, and this paper will build on existing research by meta-integrating qualitative studies of adolescents with inflammatory bowel disease (IBD), where an understanding of this experience can lead to better care for adolescents with IBD.

Population

The inclusion criteria were as follows: (a) the study population was adolescents with inflammatory bowel disease (IBD); (b) the phenomenon of interest was the experiences and experiential demands of adolescents with IBD; (c) the context of the study was after adolescents' diagnosis of IBD; and (d) the design of the study was qualitative and included, but was not limited to, phenomenological study, grounded theory, case study, historical study, and mixed study (including personal qualitative data); (e) The timeframe of the study is from the date of completion of the repository to October 2024. The exclusion criteria are as follows:

(a) full text is not available, such as conference papers and abstracts; (b) insufficient data in the articles; (c) language is not English and Chinese.

Intervention(s) or exposure(s)

Adolescent Inflammatory Bowel Disease Experiences and Feelings During the Course of the Disease. Exposure:Adolescent patients with inflammatory bowel disease

Comparator(s) or control(s)

None

Context

If the study (a) used a qualitative research design, qualitative data collection and analysis; (b) the patients were adolescents with inflammatory bowel disease, irrespective of the study setting; (c) reported the personal experiences and experiences of adolescents with inflammatory bowel disease

OUTCOMES TO BE ANALYSED

Main outcomes

Experiences and experiences of adolescents with inflammatory bowel disease.

Additional outcomes

Not applicable

DATA COLLECTION PROCESS

Data extraction (selection and coding)

Titles and abstracts of studies retrieved using the search strategy and those from additional sources will be screened independently by two review authors to identify studies that potentially meet the inclusion criteria outlined above. The full text of these potentially eligible studies will be retrieved and independently assessed for eligibility by two review authors, which will be blinded to each other's decisions. Any disagreement between the two review authors over the eligibility of particular studies will be resolved through discussion with a third review author.

Risk of bias (quality) assessment

The quality of included studies will be appraised according to the Critical Appraisal Skills Programme (CASP) checklist.

PLANNED DATA SYNTHESIS

Strategy for data synthesis
Noblit and Hare's (1988) seven step approach for synthesizing qualitative studies will be used to drive this meta-synthesis.
(a) Choosing a phenomenon to be studied.
(b) Identifying which qualitative studies are pertinent.
(c) Reading the qualitative studies to be included in the meta-synthesis.
(d) Deciding how the studies are related to one another.
(e) Each study' s metaphors are translated into the metaphors of the others and vice versa.
(f) The translations are synthesized where in a whole is created which is something more than the individual parts imply.
(g) The synthesis is expressed most often through the written word, however, plays, art, videos or music are other options.
All included studies will be read and reread to identify key metaphors. All key metaphors extracted from each study will be list on table and compared to synthesis a whole expressing. The findings of included studies will be extracted into a matrix for meta synthesis. The matrix will facilitate synthesizing and translation findings in individual study into a whole by identifying similarities and differences within and across included studies.

Analysis of subgroups or subsets
Not applicable

REVIEW AFFILIATION, FUNDING AND PEER REVIEW

Review team members

- Mr Yue Wang, Shanxi Medical University School of Medical Sciences (School of Nursing)
- Lipin Cui, Shanxi Baiqiu'en Hospital
- Xia Cheng, School of Nursing, Shanxi Medical University

Review affiliation
Shanxi Medical University School of Medical Sciences (School of Nursing)

Funding source
None

TIMELINE OF THE REVIEW

Review timeline

Start date: 09 October 2024. End date: 10 November 2024

Date of first submission to PROSPERO

09 October 2024

Date of registration in PROSPERO

19 October 2024

CURRENT REVIEW STAGE

Publication of review results

The intention is not to publish the review once completed.

Stage of the review at this submission

Review stage	Started	Completed
Pilot work	✓	
Formal searching/study identification	✓	
Screening search results against inclusion criteria	✓	
Data extraction or receipt of IP		
Risk of bias/quality assessment		
Data synthesis		

Review status

The review is currently planned or ongoing.

ADDITIONAL INFORMATION

PROSPERO version history

- Version 1.1 published on 19 Oct 2024

- Version 1.0 published on 19 Oct 2024

Review conflict of interest

None known

Country

China

Medical Subject Headings

Adaptation, Psychological; Adolescent; Humans; Inflammatory Bowel Diseases

Disclaimer

The content of this record displays the information provided by the review team. PROSPERO does not peer review registration records or endorse their content.

PROSPERO accepts and posts the information provided in good faith; responsibility for record content rests with the review team. The owner of this record has affirmed that the information provided is truthful and that they understand that deliberate provision of inaccurate information may be construed as scientific misconduct.

PROSPERO does not accept any liability for the content provided in this record or for its use. Readers use the information provided in this record at their own risk.

Any enquiries about the record should be referred to the named review contact
