# Supplementary material for: Disease experiences and perspectives of adolescent patients with inflammatory bowel disease: a meta-synthesis of qualitative research
Source: Front Public Health. 2026 Jan 13;13:1696741. doi: 10.3389/fpubh.2025.1696741 (PMC12838276; doi:10.3389/fpubh.2025.1696741)
Supplement: Supplementary file 2 [file Data_Sheet_2.docx]

**Data sheet 2. Search strategy**

**Examples of search terms - using pubmed as an example**

| Search terms | qualitative research | (qualitative research[Mesh]) OR (focus groups[Mesh]) OR (grounded theory[Mesh]) OR (anthropology，cultural[Title/Abstract]) OR (qualitative*[Title/Abstract]) OR (ethnograph*[Title/Abstract]) OR (ethnographic study[Title/Abstract]) OR (narrative research[Title/Abstract]) OR (action research[Title/Abstract]) OR (descriptive*[Title/Abstract]) OR (phenomenon*[Title/Abstract]) |
| --- | --- | --- |
|  | adolescent | “adolescent” ［Mesh］ OR “adolescent” ［Title］ OR “adolescents”［Title］ OR “adolescence”［Title］ OR “teens” ［Title］ OR “teen*” ［Title］ OR “teenager”［Title］ OR “teenagers”［Title］ OR “youth”［Title］OR “youths”［Title］ OR “juvenile”［Title］ OR “students” ［Mesh］ OR “students” ［Title］ OR adolescen* [Title/Abstract] |
|  | Inflammatory Bowel Diseases | ((Inflammatory Bowel Diseases[MeSH Terms]) OR (("Colitis, Ulcerative"[MeSH Terms]) OR (((("Colitis Gravis"[Title/Abstract]) OR ("Idiopathic Proctocolitis"[Title/Abstract])) OR ("Inflammatory Bowel Disease, Ulcerative Colitis Type"[Title/Abstract])) OR ("Ulcerative Colitis"[Title/Abstract])))) OR ((Crohn Disease[MeSH Terms]) OR ((((((((((((("Crohn's Enteritis"[Title/Abstract]) OR ("Inflammatory Bowel Disease 1"[Title/Abstract])) OR ("Regional Enteritis"[Title/Abstract])) OR (Ileocolitis[Title/Abstract])) OR ("Ileitis, Terminal"[Title/Abstract])) OR ("Terminal Ileitis"[Title/Abstract])) OR ("Ileitis, Regional"[Title/Abstract])) OR ("Regional Ileitis"[Title/Abstract])) OR ("Enteritis, Granulomatous"[Title/Abstract])) OR ("Enteritis, Regional"[Title/Abstract])) OR ("Colitis, Granulomatous"[Title/Abstract])) OR ("Granulomatous Enteritis"[Title/Abstract])) OR ("Granulomatous Colitis"[Title/Abstract])))  Filters applied: Chinese, English, from database establishment to 2024/10/1 |

**Search strategy**

**NO.1 PubMed：370**

Search time: 2024-10-1

| **Search** | **Query** | **Items found** |
| --- | --- | --- |
| #1 | (qualitative research[Mesh]) OR (focus groups[Mesh]) OR (grounded theory[Mesh]) OR (anthropology，cultural[Title/Abstract]) OR (qualitative*[Title/Abstract]) OR (ethnograph*[Title/Abstract]) OR (ethnographic study[Title/Abstract]) OR (narrative research[Title/Abstract]) OR (action research[Title/Abstract]) OR (descriptive*[Title/Abstract]) OR (phenomenon*[Title/Abstract]) | 954680 |
| #2 | “adolescent” ［Mesh］ OR “adolescent” ［Title］ OR “adolescents”［Title］ OR “adolescence”［Title］ OR “teens” ［Title］ OR “teen*” ［Title］ OR “teenager”［Title］ OR “teenagers”［Title］ OR “youth”［Title］OR “youths”［Title］ OR “juvenile”［Title］ OR “students” ［Mesh］ OR “students” ［Title］ OR adolescen* [Title/Abstract] | 2606060 |
| #3 | ((Inflammatory Bowel Diseases[MeSH Terms]) OR (("Colitis, Ulcerative"[MeSH Terms]) OR (((("Colitis Gravis"[Title/Abstract]) OR ("Idiopathic Proctocolitis"[Title/Abstract])) OR ("Inflammatory Bowel Disease, Ulcerative Colitis Type"[Title/Abstract])) OR ("Ulcerative Colitis"[Title/Abstract])))) OR ((Crohn Disease[MeSH Terms]) OR ((((((((((((("Crohn's Enteritis"[Title/Abstract]) OR ("Inflammatory Bowel Disease 1"[Title/Abstract])) OR ("Regional Enteritis"[Title/Abstract])) OR (Ileocolitis[Title/Abstract])) OR ("Ileitis, Terminal"[Title/Abstract])) OR ("Terminal Ileitis"[Title/Abstract])) OR ("Ileitis, Regional"[Title/Abstract])) OR ("Regional Ileitis"[Title/Abstract])) OR ("Enteritis, Granulomatous"[Title/Abstract])) OR ("Enteritis, Regional"[Title/Abstract])) OR ("Colitis, Granulomatous"[Title/Abstract])) OR ("Granulomatous Enteritis"[Title/Abstract])) OR ("Granulomatous Colitis"[Title/Abstract])))  Filters applied: Chinese, English, from database establishment to 2024/10/1 | 117785 |
| #4 | #1 AND #2 AND #3  Filters applied: Chinese, English,from database establishment to 2024/10/1 | 370 |

**NO.2 EMBASE：202**

Search time: 2024-10-1

| **Search** | **Query** | **Items found** |
| --- | --- | --- |
| #1 | ('inflammatory bowel disease'/exp OR (('inflammatory bowel diseases':ab,ti OR 'inflammatory bowel disease':ab,ti) AND [1988-2024]/py AND [01-01-1988]/sd NOT [02-10-2024]/sd)) AND ([chinese]/lim OR [english]/lim) AND [humans]/lim AND [embase]/lim | 185283 |
| #2 | ('ulcerative colitis'/exp OR (('chronic ulcerative colitis':ab,ti OR 'colitis ulcerativa':ab,ti OR 'colitis ulcerosa':ab,ti OR 'colitis ulcerosa chronica':ab,ti OR 'colitis, mucosal':ab,ti OR 'colitis, ulcerative':ab,ti OR 'colitis, ulcerous':ab,ti OR 'colon, chronic ulceration':ab,ti OR 'histiocytic ulcerative colitis':ab,ti OR 'mucosal colitis':ab,ti OR 'ulcerative colorectitis':ab,ti OR 'ulcerative procto colitis':ab,ti OR 'ulcerative proctocolitis':ab,ti OR 'ulcerous colitis':ab,ti OR 'ulcerative colitis':ab,ti) AND [1988-2024]/py AND [01-01-1988]/sd NOT [02-10-2024]/sd)) AND ([chinese]/lim OR [english]/lim) AND [humans]/lim AND [embase]/lim | 84676 |
| #3 | 'crohn disease'/exp AND ([chinese]/lim OR [english]/lim) AND [humans]/lim AND [embase]/lim AND [01-01-1988]/sd NOT [02-10-2024]/sd | 94638 |
| #4 | ('cleron disease':ab,ti OR 'crohn`s disease':ab,ti OR 'crohns disease':ab,ti OR 'enteritis regionalis':ab,ti OR 'intestinal tract, regional enteritis':ab,ti OR 'morbus crohn':ab,ti OR 'regional enteritis':ab,ti OR 'crohn disease':ab,ti) AND ([chinese]/lim OR [english]/lim) AND [humans]/lim AND [embase]/lim AND [01-01-1988]/sd NOT [02-10-2024]/sd | 103385 |
| #5 | #3 OR #4 | 100059 |
| #6 | #1 OR #2 OR #5 | 188737 |
| #7 | 'adolescent'/exp AND ([chinese]/lim OR [english]/lim) AND [humans]/lim AND [embase]/lim AND [01-01-1988]/sd NOT [02-10-2024]/sd | 1107376 |
| #8 | (teenager:ab,ti OR adolescent:ab,ti) AND ([chinese]/lim OR [english]/lim) AND [humans]/lim AND [embase]/lim AND [01-01-1988]/sd NOT [02-10-2024]/sd | 141149 |
| #9 | #7 OR #8 | 1132374 |
| #10 | ('qualitative research':ab,ti OR 'qualitative method':ab,ti OR 'qualitative study':ab,ti OR phenomenology*:ab,ti OR 'lived experience*':ab,ti OR hermeneutic*:ab,ti OR heideggerian:ab,ti OR husserl*:ab,ti OR 'grounded theory':ab,ti OR ethnograph*:ab,ti OR 'case study':ab,ti OR 'action research':ab,ti OR 'discourse analy*':ab,ti OR interview:ab,ti OR 'focus group*':ab,ti OR 'participant observer*':ab,ti OR 'field note*':ab,ti OR 'content analy*':ab,ti OR 'thematic analy*':ab,ti OR colaizzi:ab,ti OR giorgi:ab,ti OR 'van manen':ab,ti OR 'constant comparison':ab,ti OR 'constant comparative analysis':ab,ti) AND ([chinese]/lim OR [english]/lim) AND [humans]/lim AND [embase]/lim AND [01-01-1988]/sd NOT [02-10-2024]/sd | 344706 |
| #11 | #6 AND #9 AND #10 | 202 |

**NO.3 CINAHL (EBSCO)：40**

Search time: 2024-10-1

| **Search** | **Query** | **Items found** |
| --- | --- | --- |
| S1 | AB ( qualitative research or qualitative study or qualitative methods or interview ) AND AB ( adolescence or adolescents or teenagers or teens or youth ) AND AB ( inflammatory bowel disease or ibd or ulcerative colitis or crohn's disease )  Limiters - Publication Date: -20240931; Language: Chinese, English Interface- EBSCOhost Research DatabasesSearch Screen- Advanced Search Database- CINAHL Complete | 40 |

**NO.4 PsycINFO：31**

Search time: 2024-10-1

| **Search** | **Query** | **Items found** |
| --- | --- | --- |
| S1 | AB ( qualitative research or qualitative study or qualitative methods or interview or focus group or discussion ethnographic or mix method ) AND AB ( adolescence or adolescents or teenagers or teens or youth ) AND AB ( inflammatory bowel disease or ibd or ulcerative colitis or crohn's disease or ced or chronisch entzündliche darmkrankheiten or morbus crohn or colitis ulcerosa )  Limiters - Publication Date: -20240931; English language; Language: English  Interface- EBSCOhost Research Databases  Search Screen- Advanced Search  Database- APA PsycInfo | 31 |

**NO.5 Web of Science Core Collection：187**

Search time: 2024-10-1

| **Search** | **Query** | **Items found** |
| --- | --- | --- |
| #1 | AB=(Inflammaty Bowel Diseases OR Colitis, Ulcerative OR Colitis Gravis OR Idiopathic Proctocolitis OR Inflammaty Bowel Disease, Ulcerative Colitis Type OR Ulcerative Colitis OR Crohn Disease OR Crohn's Enteritis OR Ileocolitis OR Ileitis, Terminal OR Terminal Ileitis OR Ileitis, Regional OR Regional Ileitis OR Enteritis, Granulomatous OR Enteritis, Regional OR Colitis, Granulomatous OR Granulomatous Enteritis Granulomatous Colitis) | 53721 |
| #2 | AB=（qualitative research OR qualitative method OR qualitative study OR qualitative research OR phenomenolog* OR lived experience* OR hermeneutic* OR Heideggerian OR husserl* OR grounded theory OR ethnograph* OR case study OR action research OR discourse analy* OR interview* OR focus group* OR participant observ* OR field note* OR content analy* OR thematic analy* OR Colaizzi OR Giorgi OR Van manen OR constant comparison OR constant comparative analysis） | 3655349 |
| #3 | AB=（adolescent OR adolescent OR adolescents OR adolescence OR teens OR teen* OR teenager OR teenagers OR youth OR youths OR juvenile OR Students OR students OR adolescen*） | 613323 |
| #4 | #1 AND #2 AND #3  Search in:Select databaseWeb of Science Core Collection  Editions:Science Citation Index Expanded (SCI-EXPANDED)--1996-present | 187 |

**NO.6 Scopus：297**

Search time: 2024-10-01

| **Search** | **Query** | **Items found** |
| --- | --- | --- |
| #1 | ( TITLE-ABS-KEY ( inflammaty AND bowel AND disease ) OR TITLE-ABS-KEY ( crohn AND disease ) AND TITLE-ABS-KEY ( ulcerative AND colitis ) ) | 44573 |
| #2 | TITLE-ABS-KEY ( "qualitative research" ) OR TITLE-ABS-KEY ( "focus groups" ) OR TITLE-ABS-KEY ( "grounded theory OR anthropology&#65292;cultural" ) OR TITLE-ABS-KEY ( "qualitative*" ) OR TITLE-ABS-KEY ( "ethnograph*" ) OR TITLE-ABS-KEY ( "ethnographic study" ) OR TITLE-ABS-KEY ( "narrative research" ) OR TITLE-ABS-KEY ( "action research" ) OR TITLE-ABS-KEY ( "descriptive*" ) OR TITLE-ABS-KEY ( "phenomenon*" ) | 3861668 |
| #3 | ( TITLE-ABS-KEY ( "adolescent" ) OR TITLE-ABS-KEY ( "adolescent" ) OR TITLE-ABS-KEY ( "adolescents" ) OR TITLE-ABS-KEY ( "adolescence" ) OR TITLE-ABS-KEY ( "teens" ) OR TITLE-ABS-KEY ( "teen*" ) OR TITLE-ABS-KEY ( "teenager" ) OR TITLE-ABS-KEY ( "teenagers" ) OR TITLE-ABS-KEY ( "youth" ) OR TITLE-ABS-KEY ( "youths" ) OR TITLE-ABS-KEY ( "juvenile" ) OR TITLE-ABS-KEY ( "Students" ) OR TITLE-ABS-KEY ( "adolescen*" ) ) | 4697941 |
| #4 | #1 AND #2 AND #3 | 297 |

**NO.7 Cochrane library：61**

Search time: 2024-10-01

| **Search** | **Query** | **Items found** |
| --- | --- | --- |
| **#1** | MeSH descriptor: [Qualitative Research] explode all trees | 2492 |
| **#2** | MeSH descriptor: [Focus Groups] explode all trees | 1116 |
| **#3** | MeSH descriptor: [Grounded Theory] explode all trees | 27 |
| **#4** | ("anthropology，cultural"):ti,ab,kw OR (qualitative*):ti,ab,kw OR (ethnograph*):ti,ab,kw OR ("ethnographic study"):ti,ab,kw OR ("narrative research"):ti,ab,kw | 26642 |
| **#5** | ("action-researches"):ti,ab,kw OR (descriptive*):ti,ab,kw OR (phenomenon*):ti,ab,kw OR (interview):ti,ab,kw | 85192 |
| **#6** | #1 OR #2 OR #3 OR #4 | 103911 |
| **#7** | MeSH descriptor: [Adolescent] explode all trees | 139416 |
| **#8** | (adolescent OR adolescents OR adolescence OR teens OR teen* OR teenager OR teenagers OR youth OR youths OR juvenile OR students OR students OR adolescen*):ti,ab,kw | 225796 |
| **#9** | #7 OR #8 | 210499 |
| **#10** | MeSH descriptor: [Inflammatory Bowel Diseases] explode all trees | 5039 |
| **#11** | MeSH descriptor: [Colitis, Ulcerative] explode all trees | 2318 |
| **#12** | MeSH descriptor: [Crohn Disease] explode all trees | 2353 |
| **#13** | (Colitis Gravis OR Idiopathic Proctocolitis OR Inflammaty Bowel Disease, Ulcerative Colitis Type OR Ulcerative Colitis OR Crohn's Enteritis OR Inflammaty Bowel Disease OR Regional Enteritis OR Ileocolitis OR Ileitis, Terminal OR Terminal Ileitis OR Ileitis, Regional OR Regional Ileitis OR Enteritis, Granulomatous OR Enteritis, Regional OR Colitis, Granulomatous OR Granulomatous Enteritis OR Granulomatous Colitis):ti,ab,kw | 9704 |
| **#14** | #11 OR #12 OR #13 | 13641 |
| **#15** | #6 AND #9 AND #14  with Cochrane Library publication date to Oct 2024 | 61 |

**NO.8 CNKI：6**

**Search time: 2024-10-01**

| **Search** | **Query** | **Items found** |
| --- | --- | --- |
| #1 | TKA=“炎症性肠病 + 溃疡性结肠炎 + 克罗恩病 + IBD + CD + UC” | 83044 |
| #2 | TKA=“质性研究 + 民族志+定性研究 + 定性方法 + 现象学 + 生活经验 + 解释学 + 扎根理论 + 民族志 + 案例研究 + 行动研究 + 访谈 + 焦点小组” | 218512 |
| #3 | TKA=“青少年” | 112624 |
| #4 | #1 AND #2 AND #3 | 6 |

**NO.9 WanFang：3**

**Search time: 2024-10-01**

| **Search** | **Query** | **Items found** |
| --- | --- | --- |
| #1 | 摘要:”炎症性肠病 or 溃疡性结肠炎 or 克罗恩病 or IBD” | 67982 |
| #2 | 摘要:”质性研究 or 民族志 or 定性研究 or 现象学 or 经验 or 扎根理论 or 民族志 or 案例研究 or 行动研究 or 访谈 or 焦点小组” | 7457998 |
| #3 | 摘要:”青少年” | 229150 |
| #4 | #1 AND #2 AND #3  Date:2002-2022; Language:Chinese; Full text | 30 |

**NO.10 VIP：53**

**Search time: 2024-10-01**

| **Search** | **Query** | **Items found** |
| --- | --- | --- |
| #1 | 摘要=青少年 AND 摘要=炎症性肠病 or 溃疡性结肠炎 or 克罗恩病 or IBD AND 摘要=质性研究 or 民族志 or 定性研究 or 现象学 or 经验 or 扎根理论 or 民族志 or 案例研究 or 行动研究 or 访谈 or 焦点小组 | 53 |
