# Supplementary material for: Disease experiences and perspectives of adolescent patients with inflammatory bowel disease: a meta-synthesis of qualitative research
Source: Front Public Health. 2026 Jan 13;13:1696741. doi: 10.3389/fpubh.2025.1696741 (PMC12838276; doi:10.3389/fpubh.2025.1696741)
Supplement: Supplementary file 3 [file Data_Sheet_3.docx]

| **Data sheet 3.（Table 3：The information extraction form for the included studies.）**  Description：The content presented in this table is the detailed information of the 18 original studies included, including Author (year), Country, Study design, Data collection method, Participants, Interest/aim of the phenomenon, Findings and Main findings. So as to facilitate a clear understanding of the detailed information of the included literature. | | | | | | | | |
| --- | --- | --- | --- | --- | --- | --- | --- | --- |
|  | Author (year) | Country | Study design | Data collection method | Participants | Phenomenon of interest/Aim | Findings | Main findings |
| 1 | Allemang et al., (2024) | Canadian | Qualitative descriptive study | Virtual semistructured interviews | 21 adolescent patients with IBD aged 16 to 18 years. | To explore the mental health experiences of adolescents and young adults (AYA) with inflammatory bowel disease (IBD). | AYA with IBD endorsed the criticality of incorporating mental health discussions into routine care during the transition to adult care | Three themes were generated from the data: (1) a continuum of integration between IBD and personal identity in adolescence and young adulthood; (2) manifestations of the mind-gut connection among AYA with IBD; (3) hopes and priorities for addressing mental health in IBD care |
| 2 | Sun et al.,(2023) | China | Phenomenological research | semi-structured in-depth interview | 15 adolescent patients with IBD aged 12 to 21 years. | To understand the dilemmas experience of adolescents with inflammatory bowel disease（IBD） as they transit towards adulthood | Adolescents with inflammatory bowel disease face multiple dilemmas in preparing for the transition to adulthood. | Four themes were extracted. ①Self-coping dilemma，including difficulty in adapting to changes in the medical environment，difficulty in adapting to changes in medical roles，difficulty in coping with negative emotional distress，difficulty in meeting the special needs of daily life. ②Family support dilemma，including heavy family financial burden，excessive participation of family caregivers，and insufficient family resilience. ③Medical assistance dilemma，including limited professional assistance and limited information sharing. ④Social support dilemma，including the limited applicability and accessibility of social support，insufficient social cognition and identity. |
| 3 | Liao et al., (2023) | China | Qualitative descriptive study | semi-structure interview | 20 adolescents between the ages of 9 and 17. | To study the true feeling and coping experience of adolescent patients with inflammatory bowel disease(IBD). | Adolescents with inflammatory bowel disease (IBD) have a wide range of symptoms to cope with the disease. | Three themes of disease recognition,adaption to the change caused by diseases and self-psychological adjustment are extracted |
| 4 | Zhou and Huang, (2023) | China | Phenomenological research | semi‑structured face‑to‑face interviews | 12 adolescents between 13 and 18 years of age. | To investigate the coping mechanisms and stress perceptions of adolescent patients with Crohn’s disease. | Adolescents with Crohn’s disease can better combat the condition by implementing appropriate coping strategies. Their mental health should be given attention, and a multidisciplinary team should be assembled to pro‑ vide them with supportive care | The 2 main themes in this study were inappropriate coping mechanisms and physical and psychological stress. |
| 5 | Chen et al., (2023) | China | Qualitative descriptive study | face-to-face in-depth interviews | 14 adolescents between 14 and 17 years of age. | To explore the illness experience of adolescent patients with Crohn disease and describe the impact of the disease on the everyday lives of these individuals within the Chinese social and cultural context to provide references for targeted interventions for the healthcare team. | Healthcare providers should offer more psychological support to adolescent Crohn disease patients and advise parents to shift more attention to the mental health of their children. | Four themes were formed: (1) I am different from others, (2) I am a burden to my parents, (3) I want to be the master of my own body, and (4) I grow up suffering from illness |
| 6 | Barned et al., (2022) | Canadian | Qualitative descriptive study | semi-structured interviews | 25 paediatric patients with IBD aged between 10 and 17 years old. | To better understand Children and adolescents with Inflammatory Bowel Disease (IBD) face significant and unique challenges. | young people facing great uncertainty prior to diagnosis, pronounced changes to selfhood as they make lifestyle adjustments, and facing difficulties with the implications of reduced sociability because of their disease | Three themes: challenges related to diagnosis, making sense of change, and navigating sociability. |
| 7 | (Wu et al.,2022) | China | Descriptive phenomenology method | semi-structured interviews | 8 adolescents aged 15 to 17 years and 24 adolescents aged 18 to 25 years were included | To better investigate how AYAs experience PTG after being diagnosed | To give tailored care to patients, medical professionals must monitor the state of their PTG development in a planned and focused manner | The interviews revealed five themes: spiritual change, internalized supportiveness, cognitive re-shaping, externalized behaviors, and future-oriented thinking |
| 8 | Wang et al.,(2019) | China | Qualitative descriptive study | semi-structured interviews | 14 cases of adolescents aged 13 to 18 years. | To explore the experiences and perceptions of adolescents with inflammatory bowe disease after participating in a disease related summer camp. | The summer camp has a good effect on both disease knowledge and psychological support for adolescents with inflammatory bowel disease,thus deserving popularization. | Two themes were derived."education is the best medicine” and “the role of powerful peers” Seven sub-themeswere obtained,to improve the level of disease related knowledge, to accept the facts of illness, to increaseconfidence in treatment, to enhance the importance of self-management,to increase the sense of belonging.The summer camp has a good effect on both disto increase peer support,and to extend love. |
| 9 | Newton et al., (2019) | America | Qualitative descriptive study | semi-structure interview | 14 adolescents (12–17 years) | To explore UC experiences in general and identify any similarities and differences in the symptoms and HRQoL impacts reported by adults and/or adolescents with UC | Open-ended interviews highlighted the HRQoL and symptomatic experiences of UC from the patient’s perspective, which were similar between adult and adolescent UC patients | Only adults discussed feeling dehydrated, while only adolescents discussed the impact of UC on school life. |
| 10 | Vejzovic et al., (2018) | Sweden | Phenomenological research | Individual interviews | 7 cases of adolescents aged 10 to 18 years. | To illuminate the meaning of children’s lived experience of ulcerative colitis | Children with inflammatory bowel disease confront various problems, such as ambitions and goals that are hard to achieve, due to reduced abilities as a result of the illness or an insufficiently adapted environment | The meaning of the children’s lived experience of ulcerative colitis was summed up as a main theme. A daily struggle to adapt and be perceived as normal consisted of 4 subthemes: being healthy despite the symptoms, being healthy despite being afraid, being healthy despite a sense of being different, and being healthy despite needing support. |
| 11 | Olsen et al., (2016) | Denmark | Phenomenological research | semistructured interview | 8 cases of adolescents aged 10 to 19 years. | To identify and describe adolescents’ lived experiences while hospitalized after surgery for ulcerative colitis. | The findings demonstrate the importance of individualized nursing care on the basis of the adolescent’s age, maturity, and individual needs. | Three themes were identified: Body: Out of order; Seen and understood;and Where are all the others? |
| 12 | Barned et al., (2016) | Canadian | Qualitative descriptive study | Individual interviews | 25 cases of adolescents aged 10 to 17 years. | This study sought the perspectives of Canadian children and adolescents living with inflammatory bowel disease (IBD) to determine how they go about deciding if and when to tell others about their illness. | We argue that knowledge of how children with IBD make disclosure decisions is an important part of understanding the social experience of having IBD, and in creating environments that allow them to adapt to life with IBD. | Three themes:1.To disclose or conceal: making the decision. 2.When to tell: factors influencing disclosure decisions.3. Challenges of IBD disclosure: the reactions of others. |
| 13 | Salazar and Heyman, (2014) | California | Ethnographic study | traditional anthropological methods, such as formal and informal interviews, participant observation, and fieldnote | 25 adolescents aged 8 to 18. | The aim of the present study was to investigate knowledge of pediatric patients with inflammatory bowel disease (IBD) and perceptions of Camp Gut Busters, an IBD summer camp. | Pediatric patients with IBD who attended a disease-specific summer camp benefited from the experience. | Themes are divided into two categories:(1) Themes for attending camp:"Kids Like Me" "Not the Only One" and"Perspective on IBD";(2)Disease centered |
| 14 | Hommel et al., (2011) | America | Qualitative descriptive study | semi-structured interviews | 16 adolescents and their parents | To examine adolescent patient and parent perceptions of factors that impact adherence to IBD treatment regimens using a qualitative descriptive approach. | Patients and parents experience a number of challenges related to adherence within behavioural, educational, organisational and health belief domains. Behavioural interventions should focus on these issues, reduction of perceived barriers, and effective transition of responsibility for treatment adherence. | Three themes: Factors that impede treatment adherence； Factors that facilitate treatment adherence； Additional factors related to adherence； |
| 15 | Lynch and Spence,(2008) | New Zeeland | Phenomenological research | semi-structured interviews | 4 young New Zealanders aged between 16 and 21 years old. | To focus on discovering the youth’ thoughts, feelings, and perceptions of living with Crohn disease. | The findings reveal stress as integral to living with Crohn disease. They illuminate the paradoxical relationship between fear and hope and provide insight into what helps and what hinders young people’s ability to cope with the disease and its treatments. | Three themes: Stress as Integral to Living With Crohn Disease；The Paradoxical Relationship Between Fear and Hope； What Helps and What Hinders |
| 16 | Nicholas et al., (2007) | Canadian | Ethnographic study | depth interviews | 80 IBD participants aged between 7 and 19 years old. | To understand the lived experience and elements of quality of life as depicted by children and adolescents with inflammatory bowel disease (IBD). | Clinical assessments need to consider the experiences and perceptions of children as they manage their IBD. | Four themes: 1. Concerns relating to IBD symptoms and treatments； 2. Vulnerability and lack of control； 3. Perceiving the self negatively as different than peer； 4. Benefits of social support； 5. Personal resources in coping； |
| 17 | (Reichenberg et al., 2007) | Sweden | Grounded theory study | semi-structured interviews | 17 adolescents with IBD aged 12 to 18 years | The aim of this study was to identify, develop and relate concepts to describe the way in which adolescents experience and interpret their parents care and concern for them. | We found ambivalence to be the most distinctive theme to appear in the way in which these young people described how they felt about their parents’ response to their disease.The clinical support for young individuals with IBD should include an awareness of the simultaneous existence of conflicting attitudes, reactions and emotions. | Four main categories emerged:  ambivalence,ability/inability,compliance/resistance and trust/distrust. |
| 18 | (David et al., 2020) | America | Mixed methods | semi-structured interviews | 8 adolescents with IBD aged 12 to 20 years | To describe the expressed educational needs of pediatric patients with IBD and caregivers in regards to ostomy surgery. | Results suggest pediatric patients with IBD have limited understanding of ostomies and limited insight into educational preferences. | Five themes: Ostomy Surgery,Preoperative Concerns, Postoperative Concerns, Education Preferences,and Social Concerns, |
