# Supplementary material for: Disease experiences and perspectives of adolescent patients with inflammatory bowel disease: a meta-synthesis of qualitative research
Source: Front Public Health. 2026 Jan 13;13:1696741. doi: 10.3389/fpubh.2025.1696741 (PMC12838276; doi:10.3389/fpubh.2025.1696741)
Supplement: Supplementary file 4 [file Data_Sheet_4.docx]

**Data sheet 4. （Table 4: Thematic results of integration and example applications）**

Description: This table presents the main themes, sub-themes, and the quoted statements from the respondents that were integrated in this study.

| **Analytic themes** | **Descriptive themes** | **Quotations from participants in primary study** |
| --- | --- | --- |
| Challenges of self-remodeling and growth in adolescent patients with IBD under the influence of the condition. | The Process of Self-Cognition Reconstruction and Psychological Growth under Disease Awareness and Self-Perception | ●"I’m Not a Normal Healthy Teenager""So, in those kinds of years, it kind of puts you behind everybody else." (S-034) (Allemang et al.,2024)  ●My abdominal pain wouldn’t make me feel inferior,but the differences would … When i was diagnosed with CD, i was told to avoid strenuous exercise, so my physical strength was worse than others. Then,my grades were poor, and i felt that i was a useless person. (p12) Now, i think that this disease isn’t a very serious thing. (i) only need to take medicine and control my diet. it doesn’t have much impact (on me). (This disease) isn’t as scary as what was shown on the internet,and i just think that i could manage it on my own.(p10) (Chen et al.,2023)  ●"I admit that I have been in a negative mood at times. Compared to those in a worse situation than mine, or when I heard about some unfortunate events, I found that I was doing quite well".(Wu et al.,2022)  ●(Paddy)Once I had my surgery my attitude changed completely...I crossed over the wall. It was very hard at first...the ileostomy came as a big shock. You have to have an open mind... (Lynch and Spence,2008)  ●"I am so different because I have a strange disease"(Nicholas et al.,2007) |
|  | Complex Psychological States and Adaptive Mechanisms in Illness Management | ● This led AYA to feel isolated, “invalidated,” and “down” about “life being sick” (S-030) (Allemang et al.,2024)  ● There was several times when I said, “Let me just have the final jab and then it’s over, because I can’t take any more.” I did not want to be here any longer because I felt so bad and it just hurt. (Informant 7)(Olsen et al.,2016)  ● I think it it is kind of like umm it is not really something that you really want to talk about but it is like something it is like kind of something it is a topic you’d much rather avoid when you talk to people with you’d if I could if I I prefer not to tell very many people about it.(Barned et al.,2016)  ●(As a result of going to camp) I didn’t feel isolated. I didn’t feel like I was alone.(Salazar and Heyman,2014)  ●"I never know when I'm going to get fat again. It's awful"; You never know what is going to happen" (Nicholas et al., 2007) |
|  | Social Contextual Demands and Coping Strategies. | ●N8: ‘I'm scared to talk to my friends, my old best friends are hardly in touch now, I just want to stay at home and nobody should bother me.(Sun et al.,2023)  ●P-18: “When I knew there was no cure for IBD, my world collapsed. I locked myself in the room every day and didn’t want to face it”.(Wu et al.,2022)  ●N8:I'm fat after taking hormones, and my classmates will say I am.N10:‘My classmates make fun of me for repeating a grade and say I'm still in the fourth grade.’(Liao et al.,2023)  ●P4: "During class, I often feel compelled to go,and it’s embarrassing because I sense that my classmates are giving me odd looks whenever I do." (Zhou and Huang,2023)  ●p8:I couldn’t attend some social activities and parties.Things like communication or hanging out with my friends had become less and less frequent, and i felt as if i was estranged from my classmates to a large extent. M10:‘I just don't feel as lonely as I used to, I feel like a lot of people have this disease, I feel like I've found an organisation.(Chen et al.,2023)  ●Peggy：I have a good 5–6 friends who were always supportive. They would come with me to appointments.(Lynch and Spence,2008) |
| The role and function of family and medical systems in adolescent disease management. | Complex emotional and behavioral dynamics within family relationships of adolescent patients. | ●N1:I didn't need to prepare for the transition from paediatrics to adult unit because my parents were there and I didn't have to worry, I just listened to my parents, doctors and nurses.N5:I feel like a big pain in the arse, bothering my parents all the time, and they must hate me too, right? (Sun et al.,2023)  ●N19: Families are financially burdened and it takes a lot of time, money and effort. N2: Mum searches on the Internet and I look at what is said on the Internet with Mum. N17: Mum reads books and watches TV to learn about the disease, and I look at what I can eat with her.(Liao et al., 2023)  ●p11:I knew their intention was good, but i didn’t want to get too much attention. i felt like i was treated as“a national treasure”. i didn’t want them to act like that. I just wanted to tell them to treat me as usual; otherwise, it would impose more burden to me. (Chen et al., 2023)  ●P27: I did not know well about IBD, but my parents’ reaction to the disease scared me.(Wu et al.,2022)  ●Informant 4:“I know what a bloody nuisance one’s parents can be, but after all,they do know how their child is doing, and how they prefer things to be done, so I was extremely happy that they were with me” (Olsen et al., 2016). |
|  | Multidimensional challenges and healthcare needs experienced by adolescents in medical service encounters. | ●S-020“I feel like maybe in the future, I think [a transition navigator] is something that would benefit everyone a lot. Because there’s so many questions and things that I would not have even known about or I would have been stressed about because I wasn’t sure how to move to adult care.” (Allemang et al., 2024)  ●N8:I was hospitalised once or twice before and had to have a colonoscopy.I was scared, didn't know what it was, didn't like laxatives, and had never heard of enemas, so I was afraid and resisted.(Liao et al., 2023)  ●(Informant 7)“But those cross and cold nurses … it was mostly the older ones … they’d say ‘There, it’s like this and like that” .(Informant 4)“She was just so cold … she had no feelings whatsoever”(Olsen et al., 2016)  ●P11："I visited multiple hospitals and even consulted a Chinese medicine practitioner and tried Chinese remedies, but I didn’t experience clear improvement, and my stomach pain persisted, ultimately leading me to seek help at a hospital."(Zhou and Huang, 2023)  ●N5:I didn't know what I needed to do to prepare, or when I should start preparing, and it wasn't clear whether to pause or continue treatment when I encountered problems, and I didn't have a professional to guide me through the transition. (Sun et al.,2023)  ●‘Probably written down so I can read it and that’s how I take things in so I can look at it.’.‘I would like to have both [written and verbal] at least and also like face to face.’(David et al., 2020) |
| The intricate interplay between social contextual factors and illness adaptability in adolescents with IBD. | The Multidimensional Impact of IBD and Adolescents’ Adaptive Coping Strategies. | ●CH-012“...It’s kind of like a sharp, sharp pain...and just...It’s pain...right here on my abdomen ...That area.”(Wang et al.,2019)  ●p7:I suddenly found that i have become different.(Chen et al., 2023)  ●P3: "Undoubtedly, the greatest impact is on my academics, and frequent hospital visits eat away at my precious time.(Zhou and Huang, 2023)  ●S-027”I get stressed often, and then I do feel like I have more symptoms if I’m feeling stressed”.S-013:“In terms of IBD impacting my mood, I would say it was when I was in my flare. It took so much energy out of me, I couldn’t do anything, I was just lying in bed basically all day. (Allemang et al., 2024)  ●N9:‘The new access environment (adult healthcare system) was very different from paediatrics, [adult unit healthcare staff] were more concerned about changes in my condition and didn't approve of parental intervention, which made it difficult for me to adapt for a short period of time.’(Sun et al.,2023)  ●P2:“I know my mood is volatile; I can only do the best to adjust; watching TV is a better effective strategy to stop thinking”.P19:“Once I get into a state of self-entanglement, I will force myself not to think or find something else to do to divert my attention”P8:“I want to make myself feel better. Because I found that mood is magical,when I am in a good mood, my bowel symptoms will improve accordingly. My doctor also told me that they were related”.P20: “I used to put my career first, giving up my health for work. It’s not too late to realize that health is the most important thing”(Wu et al.,2022) |
|  | Social Environmental Barriers and Resource Utilization Patterns. | ●N4:‘Sometimes I have to poop dozens of times in 1 d. I often go to places where there are not always bathrooms, and I can't get there in time when the urge to poop comes.’ N13:‘Biological agents need to be used regularly, but they are more expensive, with high out-of-pocket expenses, there is no way to buy commercial insurance for this disease, there is no corresponding supportive policy, and I have limited social support. ’(Sun et al.,2023)  ●And I didn’t really how to handle between that and school.” (S-031) .“When I was sick it was very frustrating, not being able to go out or being too scared to go out. I remember a couple of times I went to [amusement park] with my family and I wasn’t even able to make it to the front of the park without needing to go to the bathroom. I remember my dad had to carry me and as a little bit of an older kid, I was in the fifth or sixth grade, but it’s a little embarrassing I’m not gonna lie. It’s definitely hard.” (S-030) (Allemang et al.,2024)  ●AIDS everybody knows about. But when have you seen a commercial that says‘help people with ulcerative colitis’？(Salazar and Heyman, 2014)  ●"I'm thinner, pale, my hair is thinner. I don't like how I look now. I look sickly"; "Everyone else is growing, but I'm shrinking"; (Nicholas et al., 2007)  ●They put me on prednisone and I had a reaction. It made my whole body blow up. I put on so much weight and my face went all round…. It changes your whole look. （Amy）.I was really scared of coming out with a bag.... I love swimming and surfing at the beach so that was huge worry.... I still have ongoing crap ... the usual toilet dramas, but not as often. (Amy).(Lynch and Spence,2008) |
